# Prevalence, Antimicrobial Resistance Profiles, Virulence and Enterotoxins-Determinant Genes of MRSA Isolated from Subclinical Bovine Mastitis in Egypt

**DOI:** 10.3390/pathogens9050362

**Published:** 2020-05-09

**Authors:** Abdelazeem M. Algammal, Mohamed E. Enany, Reham M. El-Tarabili, Madeha O. I. Ghobashy, Yosra A. Helmy

**Affiliations:** 1 Department of Bacteriology, Immunology and Mycology, Faculty of Veterinary Medicine, Suez Canal University, Ismailia 41522, Egypt; enanyeg@yahoo.com (M.E.E.); rehameltrabely@gmail.com (R.M.E.-T.); 2 Microbiology Department, Faculty of Science, Ain Shams University, Cairo 11556, Egypt; Mghobashy@ut.edu.sa; 3 Biology Department, Faculty of Science, Tabuk University, Tabuk 71491, Saudi Arabia; 4 Food Animal Health Research Program, Department of Veterinary Preventive Medicine, Ohio Agricultural Research and Development Center, The Ohio State University, Wooster, OH 44691, USA; 5 Department of Animal Hygiene, Zoonoses and Animal Ethology, Faculty of Veterinary Medicine, Suez Canal University, Ismailia 41522, Egypt

**Keywords:** MRSA, subclinical bovine mastitis, prevalence, antimicrobial resistance, virulence genes, enterotoxins genes

## Abstract

Subclinical mastitis caused by *Staphylococcus aureus* has worldwide public health significance. Here, we aimed to determine the prevalence of *S. aureus*, antimicrobial resistance profiles, and the virulence and enterotoxins determinant genes of MRSA strains that caused subclinical bovine mastitis. Milk samples were collected from 120 lactating animals (50 buffaloes and 70 dairy cattle) from different farms located in Ismailia Province (Egypt). The collected samples were investigated for subclinical mastitis using a California mastitis test. The total prevalence of *S. aureus* was 35.9% (84/234) with 36.3% (53/146) in cattle and 31% (31/88) in buffaloes. Antimicrobial susceptibility testing showed that 35.7% (30/84) of the isolated strains were resistant to cefoxitin, defined as methicillin-resistant *S. aureus* (MRSA), with 37.7% (20/53) in cattle and 32.2% (10/31) in buffaloes. Using PCR, 100% of the tested strains harbored *coa* and *mecA* genes, while 86.6% were positive for *spa* gene, with remarkable gene size polymorphism. Additionally, 10% of the tested strains contained the *pvl* gene. Further, using multiplex PCR, 26.6% of the tested samples had *sea* gene, two strains had *sec* gene and only one strain had *sea* and *sec* genes. The *seb* and *sed* genes were absent in the tested strains. In conclusion, *mecA, coa* and *spa* virulence genes were widely distributed in MRSA strains isolated from bovine milk, whereas the *sea* gene was the most predominant enterotoxin gene. Notably, this is the first report that emphasizes the prevalence of *pvl* gene of MRSA isolated from bovine milk in Egypt.

## 1. Introduction

*Staphylococcus aureus* is a significant public health bacterial pathogen, causing mastitis in dairy animals including cattle, buffalo, sheep and goats [1]. *S. aureus* mastitis and its produced toxins lead to great economic losses in dairy farms due to: (1) reduction in the milk production, (2) alteration in the composition and quality of the produced milk, (3) the need to discard the produced milk, (4) early culling of infected animals, and (5) high cost of treatment and control [2]. Resistance of *S. aureus* to several antimicrobials complicates the treatment of these pathogenic bacteria, which is considered an increasing challenge. Methicillin-resistant *S. aureus* (MRSA) strains can cause nosocomial infections and consequently high mortality in humans [3]. In Egypt, there was high prevalence of resistance among *S. aureus* in bovine species to antimicrobial agents such as β-lactams, which are used to treat mastitis [4,5]. This high prevalence is caused by the uncontrolled widespread use of antibiotics. Therefore, MRSA has high clinical significance and poses a potential public health hazard.

The ability of *S. aureus* to cause infections is due to virulence factors, such as the secretion of several toxins and presence of cell wall adhesion proteins. Thus the bacteria can survive in the udder, causing chronic inflammation [6]. Coagulase is one of the *S. aureus* virulence factors that stimulates prothrombin, resulting in blood clotting [7,8]. Additionally, protein A is a cell wall component that hinders phagocytosis by neutrophils and contains the Fc-portion, X-segment, and C-terminal portion. This X-region of *spa* gene usually undergoes repetition (up to 24 repeats) and differs from one strain to another [9,10]. Another virulence factor of *S. aureus* is leukotoxin which is very toxic to WBCs, especially neutrophils. The most important *S. aureus* leukotoxin is Panton-Valentine leukocidin (PVL) which is composed of S and F proteins and destroys the neutrophil’s cell membrane. PVL and SEs are the most potent virulence determinants of *S. aureus* with a significant role in the initiation and pathogenesis of the disease [11,12].

Milk ingredients enhance the growth of *S. aureus* and subsequently the production of enterotoxins which are heat stable and resist pasteurization. Therefore, raw milk with improper storage standards has an increased rate of food intoxication. For example, *S. aureus* enterotoxin A, considered as a potent virulence markers, can resist heating temperature up to 121 °C for 20 min [13]. Further, staphylococcal enterotoxins (A, B, C, D, and E) are the primary cause of food poisoning outbreaks, while the other types are responsible for sporadic cases [8,14].

The estimated population of cattle and buffaloes in Egypt by 2019 was 9.3 million head, with more than 7.2 million tons of milk production [15]. Therefore, in this study, we aimed to investigate the prevalence and antimicrobial resistance of *S. aureus* in tested milk samples, and investigate the prevalence virulence determinant (*coa, spa, pvl* and *mec*A) and enterotoxins *(sea*, *seb*, *sec* and *sed*) genes of MRSA isolated from bovine species milk in Egypt.

## 2. Materials and Methods 

### 2.1. Collection of Milk Samples

Four hundred eighty milk samples were collected in the period between December 2018 and February 2019 under aseptic conditions. Milk samples were collected from 120 clinically healthy lactating animals from two farms (50 local breed buffaloes from one farm and 70 dairy cattle from the other farm) located in Ismailia Province, Egypt. The selected farms use manual milking regimes and practice intensive management systems. Four milk samples were collected from each animal (one sample per quarter of the udder).

Subclinical mastitis animals were selected based on specific characteristics including: (1) being apparently unaffected by the illness, and (2) exhibiting a reduction in milk yield, which might result in high somatic cell count.

The handling of animals was done as described by the Animal Ethics Board Committee of Suez Canal University, Egypt. Before sample collection, the udder of each animal was palpated for the detection of any abnormalities such as swelling, hotness, asymmetry, and any physical changes. Animal’s udder, teats, and hands of the examiner were washed using running water and soap and were dried with a clean towel. The udder, teats, and tester hands were then sterilized with 70% ethyl alcohol to ensure that there was no external contamination. The first strips of milk were excluded and thrown away as they may be contaminated from the teat orifice, then 15–20 mL of milk samples were collected from each quarter into sterile screw-capped McCartney bottles (Thermo Fisher Scientific, Waltham, MA, USA). Milk samples were immediately transported to the laboratory in an ice container [16].

### 2.2. California Mastitis Test (CMT)

In order to determine the milk samples infected with subclinical mastitis, CMT (screening test) was used. The test procedures were conducted as previously mentioned [17,18]. The test depends on the interaction of the reagent with DNA of somatic cells present in milk. Briefly, in a cup of the white plastic paddle, 2 mL of scam reagent was added to an equal volume of milk sample from each quarter of the udder and mixed by a gentle circular motion. The test results were evaluated visually within 20 s and interpreted as; − (0), ± (T), + (1), + + (2), and + + + (3) based on the amount of gel formation. Milk samples from individual quarters with positive CMT scores were subjected to bacteriological examination.

### 2.3. Isolation and Identification of S. aureus

The screened positive milk samples were incubated at 37 °C for 24 h, and then centrifuged at 3000 rpm for 5 min. The cream layer was discarded and sediments were streaked onto blood agar, nutrient agar, and mannitol salt agar plates (Oxoid, Hampshire, UK). The streaked plates were then incubated at 37 °C for 24–48 h. The suspected grown colonies were identified morphologically and biochemically as previously mentioned [17]. *S. aureus* circular convex golden-yellow colonies were collected and preserved at −80 °C in media containing 10% glycerol (*v*/*v*) for further analysis. The confirmation of the retrieved colonies was performed using PCR for 16Sr RNA gene identification as previously described [19].

### 2.4. Antimicrobial Susceptibility Testing of S. aureus

Antimicrobial susceptibility testing was carried out using disc diffusion technique [20]. The isolated strains were tested for their susceptibility to cefoxitin (Cef; indicative for MRSA), penicillin (Pen), ampicillin-sulbactam (Amp-Sul), amoxicillin-clavulanic acid (Amo-Cla), tetracycline (Tet), cefotaxime (Ceft), and erythromycine (Ery) (Oxoid).

The selected antimicrobials are representative of the drugs used for humans and in the animal industry and were chosen according to the National Antimicrobial Resistance Monitoring System (NARMS) records. The reference strain (*S. aureus* ATCC 25923) was used as a control for the disc diffusion technique. The test was conducted on Muller Hinton agar plates (MH, Oxoid) and the plates were incubated at 37 °C for 24 h. The test was performed in accordance with the recommendations of the Clinical Laboratory Standards Institute (CLSI) criteria using the available CLSI interpretive criteria (Table 1).

### 2.5. Detection of Virulence and Enterotoxins Genes of MRSA Strains Using PCR

#### 2.5.1. Genomic DNA Extraction

DNA of MRSA strains was extracted using the boiling method. Briefly, a half loopfull from *S. aureus* plate cultures were suspended in 100 μL of DNase-free water, heated at 95 °C for 10 min, cooled, and then centrifuged at 5000× *g* for 10 min. The supernatant containing the genomic DNA was collected in a new tube and stored at −20 °C for further use. DNA was quantified using a Nanodrop 1000 instrument (Thermo Scientific, Loughborough, UK).

#### 2.5.2. Polymerase Chain Reaction (PCR)

The extracted DNA from MRSA strains were screened for virulence genes (*coa, spa, pvl and mecA*) and enterotoxins genes *(sea, seb, sec* and *sed*) detection. Amplification was performed in PCR tubes, in a 25 µL reaction volume, containing 200 µM of dNTPS buffer (dATP, dGTP, dCTP and dTTP), 50 picomol of each forward and reverse primers, and 0.5 units of taq DNA polymerase (NZYtech, Lisbon, Portugal). The PCR reaction mixtures were amplified in the MJ MiniTM Gradient Thermocycler apparatus (Biometra, Göttingen, Germany). Primers sequence, expected amplicon size, and annealing temperature are described in Table 2. Nuclease-free water was used as a negative control. Positive controls DNA were obtained from the Department of Microbiology, Faculty of Veterinary Medicine, Suez Canal University, Egypt. PCR products were visualized on a 1.5% agarose gel containing ethidium bromide under UV light and 100 bp ladder (Fermentas, Thermo Scientific, Darmstadt, Germany) was used.

### 2.6. Statistical Analysis

The Chi-square test was used for the analysis of the recovered frequencies using SAS^®^ software (version 9.4, SAS Institute, Cary, NC, USA) to test the null hypothesis of various treatments. A *p*-value of <0.05 was considered statistically significant.

## 3. Results

### 3.1. Prevalence of S. aureus Subclinical Bovine Mastitis

The prevalence of subclinical mastitis in the collected milk samples from individual quarters using CMT was 44% (88/200) and 52.1% (146/280) in buffaloes and cattle, respectively (Table 3). There was no significant differences between the prevalence of subclinical mastitis between buffaloes and cattle (*p* > 0.05). Additionally, the total prevalence of *S. aureus* in the collected milk samples from individual quarters using CMT was 35.9% (84/234) with 36.3%% (53/146) in cattle and 31% (31/88) in buffaloes and no significant difference between cattle and buffaloes (*p* = 0.8654; X^2^ = 0.029). Out of them, the prevalence of MRSA was 35.7% (30/84) with 37.7% (20/53) in cattle and 32.2% (10/31) in buffaloes and no significant difference between cattle and buffaloes (*p* = 0.6203; X^2^ = 0.245). Detailed results of CMT screening in the collected milk samples are shown in Table 4.

### 3.2. Antimicrobial Susceptibility Phenotypic Profiles of the S. aureus Isolates

The antimicrobials susceptibility test for *S. aureus* strains showed that 64.3% (54/84) of the samples were resistant to penicillin, 59.5% (50/84) were resistant to tetracycline and 35.7% (30/84) of the strains were resistant to cefoxitin (defined as MRSA). In addition, 58.3% (49/84) of the tested strains exhibited intermediate sensitivity to cefotaxime, whereas 64.3% (54/84) of the tested strains were sensitive to cefoxitin. Our results also showed that 78.6% (66/84) of the strains were sensitive to amoxicillin-clavulanic acid, 72.6% (61/84) to ampicillin-sulbactam, and 63.1% (53/84) were sensitive to erythromycin. Details about the antimicrobial susceptibility phenotypic profiles of the *S. aureus* strains are shown in Table 5.

### 3.3. Virulence Determinant Genes of MRSA Strains

Thirty isolates of MRSA strains were subjected to PCR for the detection of *coa*, *spa*, *pvl* and *mec*A genes. All the 30 tested strains (100%) were positive for *coa* gene that showed no gene polymorphism, while 26 out of 30 strains (86.6%) were positive for *spa* gene and showed a remarkable gene polymorphism with different amplicons size (140 bp, 270 bp and 290 bp). Only 3 out of 30 strains (10%) were positive for *pvl* gene. Furthermore, all the examined strains (100%) were positive for *mec*A gene. There is a significant difference in the prevalence of virulence determinant genes (*p* < 0.0001) among the examined MRSA strains (Table 6).

### 3.4. Prevalence of Enterotoxins Genes among MRSA Strains

The prevalence of enterotoxins genes (*sea, seb, sec* and *sed*) among MRSA strains were detected using multiplex PCR. Our results showed that eight out of 30 strains (26.6%) were positive for *sea* gene, two out of 30 strains were positive for *sec* gene, one out of 30 strains (3.3%) was positive for both *sea* and *sec* genes, and none of the tested strains harbored *seb* and *sed* genes. Hence, 11 out of 30 strains (36.6%) were enterotoxignic (Table 6). The prevalence of the enterotoxins genes showed a significant difference (*p* < 0.0001) among the examined MRSA strains.

## 4. Discussion

Subclinical mastitis is a significantly important disease, causing economic losses in the livestock industry, not only in Egypt, but also worldwide. Antibiotic resistance has increased among various bacterial pathogens, which is considered an emerging problem with a major public health concern due to the risk of resistance transmission to human as well as its influence on the effectiveness of the current antibiotic therapy [26,27,28,29]. Further, MRSA strains can cause nosocomial infections and high mortality in humans [3]. In this study, the detected prevalence of subclinical mastitis was 44% and 52.1% in buffaloes and cattle, respectively, with a total prevalence of 48.6% and no significant difference in the prevalence between both animal species (Table 3). This high prevalence of bovine subclinical mastitis was similar to the results obtained in other previous studies [16,30]. The increased incidence of subclinical mastitis in dairy livestock is attributed to multiple predisposing factors including: (1) contaminated milking machines, (2) improper housing, (3) bad sanitation, and (4) bad handling of animals. Furthermore, the failure in the treatment always occurred due to: (1) chronic infection accompanied with fibrosis, (2) inadequate dose of antibiotics, and (3) emergence of multidrug-resistant bacterial pathogens [31].

The prevalence of *S. aureus* recovered from subclinical mastitis infected animals was 36% (*p* < 0.0001). This result was similar to previous studies [16,32] and lower than the prevalence (6.5%) reported by Haltia, et al. [33]. *S. aureus* transmission between animals is due to using of contaminated milk utensils and is also due to contaminated milker’s hands [34]. Additionally, cefoxitin resistance (MRSA) was used determine the methicillin-resistant *S. aureus* isolates, and antimicrobial susceptibility testing showed that 35.7% of the recovered *S. aureus* strains were resistant to cefoxitin (Table 5). Further, the identified MRSA strains were confirmed using PCR for detection of *mec*A gene, where all the tested strains (100%) harbored this gene (Table 6) conferred marked resistance to various antimicrobial agents such as cefoxitin, penicillins, cephalosporins, macrolides, aminoglycosides and tetracyclines. Since the 1990s, most MRSA isolates possessed a multidrug-resistant phenotype and carried many resistant determinants in chromosome and plasmids. Resistance to methicillin is attributed to the existence of *mecA* gene on the *S. aureus* chromosome, which is encoded for the synthesis of PBP_2a_. Methicillin is stable in the presence of β-lactamase enzymes and is effective in the treatment of *S. aureus* infection, but not against MRSA that resist methicillin [35,36,37].

In this study, 64.3% *S. aureus* strains were resistant to penicillin which was in agreement with other studies (more than 50%) [38] and lower than resistance rate (7.1%) determined by Bengtsson, et al. [39]. The failure of treatment of subclinical mastitis with penicillin was mainly due to the release of β-lactamase by *S. aureus*, which causes hydrolysis of β-lactam rings. The majority of *S. aureus* strains release penicillinases which consequently make *S. aureus* resistant to the β-lactam group of antibiotics [40]. Furthermore, 78.57% *S. aureus* strains were sensitive to amoxicillin-clavulanic acid, while 72.62% was sensitive to ampicillin-sulbactam. The isolation of a naturally occurring β-lactamase inhibitor (clavulanic acid) from *Streptomyces clavuligerus* was a major step in the formulation of new antibiotic combinations. Sulbactam and clavulanic acid (β-lactam antibiotics) have very poor bactericidal ability, however, they are a very strong β -lactamase inhibitors [41].

We observed that 59.5% of the strains were resistant to tetracyclin, a broad-spectrum antibiotic groups that, due to its extensive use, resulted in the development of resistant strains. Resistance to tetracyclines resulted from the production of ribosomal protection protein by *S. aureus* that makes a competitive binding to tetracycline. The *tet* gene that is carried on conjugative plasmids of *S. aureus* is responsible for their production [42].

In addition, 58.3% of the *S. aureus* strains were moderately sensitive to cefotaxime, while 63.1% were sensitive to erythromycin. In general, third-generation cephalosporins have strong activity against Gram-negative bacteria and moderate activity against Gram-positives, such as *S. aureus* and *Streptococci*. Unlike penicillin, third-generation cephalosporins have stable activity in presence of β-lactamase enzyme. Erythromycin belongs to the macrolide class and showed a potent antibacterial activity against both Gram-positive and Gram-negative bacteria, including *Staphylococci*, *Streptococci* and *E. coli* [41,43].

The *coa* gene was 100% prevalent in the tested MRSA strains. These results are similar to those reported by Akineden, Annemüller, Hassan, Lämmler, Wolter and Zschöck [24]. This gene exhibited no size polymorphisms [44]. However, in other studies, coagulase gene amplification resulted in different amplicons, indicating coagulase gene size polymorphism [34]. The *spa* gene displayed remarkable gene polymorphisms where different sized amplicons were found (140, 270, and 290 bp). The X region of *spa* gene usually undergoes variable repetitions (up to 24 repeats) which might be different in different strains [10]. Number of repeats is associated with the dissemination potential of *S. aureus,* where strains that have more than seven repeats in the X region were considered as epidemic, whereas the presence of seven or less repeats were considered as non-epidemic MRSA [14]. Further, the *pvl* gene was detected in 10% of the tested strains. The presence of *pvl* gene in *S. aureus* was similar to that obtained previously [45] and disagrees with the results obtained by Ikawaty, et al. [46]. The *pvl* gene is considered as the most powerful staphylococcal leukotoxin that could resist bovine neutrophils [47]. Therefore, *pvl* may contribute to resistance by attacking the bovine polymorph-nuclear cells and increase pathogenicity against the host [46]. Interestingly, the *sea* gene was detected in 26.6% of the tested strains followed by *sec* gene (6.6%) then mixed (*sea* and *sec*) genes in 3.3%, while none of MRSA strains harbored *seb* and *sed* genes (Table 6). The high prevalence of *sea* and *sec* gene was previously reported by Rall, et al. [48]. The *sea* gene was the most predominant enterotoxins gene isolated in other studies conducted on *S. aureus* [49]. This gene is very resistant to pasteurization heat and maintain some biological activity after 28 min at 121 °C [50]. It’s also considered as the most frequently detected gene in the US food poisoning outbreaks followed by *sed* and *seb* and 95% of these outbreaks have *sea* and *see* enterotoxins [51].

## 5. Conclusions

In conclusion, subclinical mastitis caused by *S. aureus* is considered as one of the major economically important diseases with public health significance. The most predominant virulence genes associated with MRSA strains in bovine milk were *coa*, *mec*A and *spa*, whereas, the most predominant enterotoxin gene is *sea* that causes food poisoning in human consumers after ingestion of contaminated milk. Based on our knowledge, this is the first study that emphasizes the occurrence of *pvl* in MRSA strains originating from bovine species milk in Egypt. The continuous application of the antimicrobial susceptibility testing of *S. aureus* in the future will be necessary to determine the drug choice for disease control.

## Figures and Tables

**Table 1:** Interpretive criteria for inhibition zone diameter [20,21].

Antimicrobial Agent	Disc Conc.	Diameter of Inhibition Zone (mm)		
		R	I	S
Pen	10 units	28 or less	-	29 or more
Amo-Cla	10–20 µg	19 or less	-	20 or more
Amp-Sul	10 µg	11or less	12–14	15 or more
Tet	30 µg	14 or less	15–18	19 or more
Ceft	30 µg	14 or less	15–22	23 or more
Cef	30 µg	≤21 mm	-	-
Ery	15 µg	13 or less	14–17	18 or more

R: Resistant, I: Intermediate, S: Sensitive. According to the National Committee for Clinical Laboratory standards. Cefoxitin (Cef); Penicillin (Pen); Ampicillin-sulbactam (Amp-Sul); Amoxicillin-clavulanic acid (Amo-Cla); Tetracycline (Tet); Cefotaxime (Ceft); Erythromycine (Ery).

**Table 2:** List of primers and recycling conditions of PCR assay.

Primer	Primer Sequence.	Annealing Temperature	Recycling Conditions	References
*coa1*	ATA GAG ATG CTG GTA CAG G	58 °C	39 cycles; 94 °C for 1 min, 58 °C for 1 min, 72 °C for 1 min	[22]
*coa 2*	GCT TCC GAT TGT TCG ATG C			
*sea-3b*	CCT TTG GAA ACG GTT AAA ACG	55 °C	30 cycles; 95 °C for 1 min, 55 °C for 1 min, 72 °C for 2 min	[23]
*sea-4b*	TCT GAA CCT TCC CAT CAA AAA C			
*seb-1c*	TCG CAT CAA ACT GAC AAA CG	55 °C		
*seb-4b*	GCA GGT ACT CTA TAA GTG CCT GC			
*sec-3b*	CTC AAG AAC TAG ACA TAA AAG CTA GG	55 °C		
*sec-4b*	TCA AAA TCG GAT TAA CAT TAT CC			
*sed-3b*	CTA GTT TGG TAA TAT CTC CTT TAA ACG	55 °C		
*sed-4b*	TTA ATG CTA TAT CTT ATA GGG TAA ACA TC			
*spa-III*	CAA GCA CCA AAA GAG GAA	60 °C	30 cycles; 94 °C for 1 min, 60 °C for 1 min, 72 °C for 1 min	[24]
*spa-IV*	CAC CAG GTT TAA CGA CAT			
*luk-PV-1*	ATCATTAGGTAAAATGTCTGGACATGATCCA	66 °C	34 cycles; 94 °C for 30 s, 66 °C for 30 s, 72 °C for 1 min 30 s	[25]
*luk-PV-*2	GCATCAACTGTATTGGATAGCAAAAGC			
*mecA-1*	TGGCATTCGTGTCACAATCG	53 °C	34 cycles; 94 °C for 1 min, 53 °C for 50 s, 72 °C for 1 min	[25]
*mecA-2*	CTGGAACTTGTTGAGCAGAG			

**Table 3:** Prevalence of subclinical mastitis in buffaloes and cattle (based on CMT).

Animal Species	Total Animals (No.)	Total Samples (No.)	Negative Samples (No.)	Positive Samples (No.)	Positive Samples (%)	Chi-Square Value
Buffaloes	50	200	112	88	44	3.057 NS^ *^ p = 0.0804
Cows	70	280	134	146	52.1	
Total	120	480	246	234	48.75	

* NS = non-significant.

**Table 4:** Results of CMT screening in the collected milk samples.

CMT Grads	Examined Quarters (No.)	Positive *S. aureus* Isolates (No.)	*S. aureus* Isolates (%)
+++	57	43	75.4
++	39	32	82
+	138	9	6.5
Total	234	84	35.9

Percentages were calculated in comparison to the total number of examined samples of each grade.

**Table 5:** Antimicrobial sensitivity test of the isolated *S. aureus* strains.

Antimicrobial Agents	Resistant		Intermediate		Sensitive	
	No.	%	No.	%	No.	%
Cef	30 (MRSA)	35.7	-	-	54	64.3
Pen	54	64.3	-	-	30	35.7
Amo- Cla	18	21.4	-	-	66	78.6
Amp-Sul	11	13.1	12	14.3	61	72.6
Tet	50	59.5	15	17.9	19	22.6
Ceft	13	15.5	49	58.3	22	26.2
Ery	26	30.9	5	5.9	53	63.1
Chi-square value	94.7860 * p < 0.0001		186.19 * p < 0.0001		104.25 * p < 0.0001	

* Significant differences in the prevalence between the antimicrobial agents. C*ef*oxitin *(*Cef); Penicillin (Pen); *Amp*icillin-*sul*bactam (Amp-Sul); A*mo*xicillin-*cla*vulanic acid (Amo-Cla); *Tet*racycline (Tet); *Cef*o*t*axime (Ceft); E*ry*thromycin *(*Ery). There was significant differences between Cef and Pen: X^2^ = 13.660, *p* = 0.0002, Cef and Amp-Sul: X^2^ = 11.560, *p* = 0.0007; Cef and Tet: X^2^ = 9.481, *p* = 0.0021; Pen and Amo-Cla: X^2^ = 31.376, *p* < 0.0001; Pen and Amp-Sul: X^2^ = 46.134, *p* < 0.0001; Pen and Ceft: X^2^ = 41.462, *p* < 0.0001; Pen and Ery: X^2^ =18.673, *p* < 0.0001; Amo-Cla and Tet: X^2^ = 25.160, *p* < 0.0001; Amp-Sul and Tet: X^2^ = 38.873, *p* < 0.0001; Tet and Ceft: X^2^ = 34.487, *p* < 0.0001; and Tet and Ery: X^2^ = 13.787, *p* = 0.0002.

**Table 6:** Prevalence of virulence and enterotoxins determinant genes of MRSA strains isolated from bovine milk.

Genes		No	%	Chi-Square Value
Virulence Genes	*coa*	30	100	62.6900 * p < 0.0001
	*spa*	26	86.6	
	*pvl*	3	10	
	*mec*A	30	100	
Enterotoxins Genes	*sea*	8	26.6	21.9751 * p < 0.001
	*sea+sec*	1	3.3	
	*sec*	2	6.6	
	*seb*	0	0	
	*sed*	0	0	
Chi-square value		168.0403 * p < 0.0001		

No of the examined MRSA strains= 30. There were significant differences between *spa* and *pvl* at X^2^ = 34.659, between *coa* and *pvl* at X^2^ = 48.273; and between *mecA* and *pvl* virulence genes at X^2^ = 48.273 (*p* < 0.0001). There were significant differences between *sea* and *seb/sed* enterotoxins genes at X^2^ = 9.051 and *p* = 0.0026. * Significant difference in the prevalence between different virulence genes and between different enterotoxins genes.
